# Orbital apex syndrome secondary to myocysticercosis: A case report from Nepal

**DOI:** 10.1016/j.amsu.2022.104336

**Published:** 2022-08-05

**Authors:** Bipin Koirala, Sangam Shah, Sanjeeta Sitaula, Gulsan Bahadur Shrestha

**Affiliations:** a Himalaya Eye Institute, Pokhara University, Nepal; b Maharajgunj Medical Campus, Institute of Medicine, Tribhuvan University, Maharajgunj, 44600, Nepal; c Department of Ophthalmology, Maharajgunj Medical Campus, Institute of Medicine, Tribhuvan University, Maharajgunj, 44600, Nepal

**Keywords:** Cysticercosis, Orbital apex syndrome, Nepal

## Abstract

**Introduction:**

Cysticercosis is caused by parasitic infestation mainly by the larval form of *Taenia solium.* Orbital cysticercosis may involve both the intraocular structures and orbit particularly the extraocular muscles. The clinical manifestations are caused mainly by the mass effect of the cyst in the initial period resulting in ocular motility restriction and proptosis and depends primarily on the site of the lesion.

**Case presentation:**

Here we report a case of 27 years old male with orbital apex syndrome secondary to myocysticercosis.

**Discussion:**

Orbital myocysticercosis often mimics various eye pathologies like as isolated nerve palsy, orbital pseudotumor, orbital cellulitis. Acute vision loss in a case of orbital cysticerosis is mainly due to compressive optic neuropathy due to cystic lesion of extra ocular muscle or by direct invasion of the optic nerve. In our case, MRI orbit revealed cysticercosis of lateral rectus at orbital apex where it compressed the optic nerve resulting compressive optic neuropathy. Albendazole along with steroid was used as the first-line treatment.

**Conclusion:**

This case highlights that Orbital Apex Syndrome Secondary to Myocysticercosis is a rare and can lead to severe visual loss if not managed timely.

## Highlights

• Cysticercosis is serious health problem in many developing countries of Asia like Nepal.
• Orbital cysticersosis can be sight threatening so early diagnosis and intervention can prevent vision loss.

## 1 Introduction

Cysticercosis is the most common parasitic disease affecting the nervous system of humancaused by parasitic infestation mainly by the larval form of *Taenia solium*. It is a serious health problem in many developing countries of Asia, Africa and Latin America where there is poor sanitation and improper management of food and meat products [1]. Cysticerci travel via the hematogenous route and usually lodge themselves at sites with high glycogen content [1]. Orbital cysticercosis may involve both the intraocular structures and orbit particularly the extraocular muscles [2]. Clinical features commonly encountered in Orbital Cysticercosis are extraocular motility limitations, retro-bulbar resistance, swollen lids, conjunctival congestion, strabismus, ptosis, and rarely, decrease in vision. The clinical manifestations are caused mainly by the mass effect of the cyst in the initial period resulting in ocular motility restriction and proptosis and depends primarily on the site of the lesion. There is no any predilection for a particular extra ocular muscle for occurring cysticercosis, as the commonly involved muscle varies according to different literature. Here we report a case of 27 years old male with orbital apex syndrome secondary to myocysticercosis. This case has been reported as per SCARE 2020 criteria [3].

## 2 Case presentation

A 27 years old male presented to our center with chief complaints of sudden onset of binocular double vision which increases towards left side gaze along with mild periorbital pain for 3 days accompanied by painless rapidly progressive deterioration of vision in the left eye over 1 day. He had no history of trauma, fever, headache, vomiting, neck stiffness, insect bite, rashes, upper respiratory tract infections, ear discharge or decreased hearing. He had no hypertension, diabetes, and history of hyperlipidemia. He doesn't smoke or consumes alcohol and is non-vegetarian by diet.

**Fig. 1:**
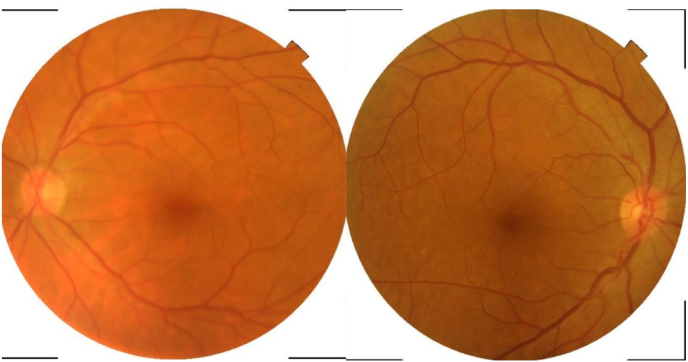
(a) and (b): Early disc edema with obliterated CDR in left eye and normal right eye.

On examination, best corrected visual acuity (BCVA) in the right (RE) and left eye (LE) was 6/6 and 6/60 respectively. LE had 15 prism diopter esotropia with marked limitation of abduction. Motility of right was normal on duction testing. His face was turned on left side (Fig. 1). Pupils were round reactive in both eyes measuring 3–4 mm. Swinging flash light test revealed grade 2 Relative Afferent Pupillary Defect (RAPD)in left eye. Anterior segment and vitreous findings were normal in both eyes with clear cornea and normal corneal sensation. Posterior segment examination revealed normal fundus findings in right eye and with signs of early disc edema, obliterated C/D ratio, normal macula and vessels in left eye (Fig. 1). On examination of cranial nerves (CN), CN II and CNVI was impaired while CN III, IV, V1, V2, VII-XII were grossly intact. Motor and sensory functions of bilateral upper and lower extremities were grossly intact.

**Figure (2a, 2b):**
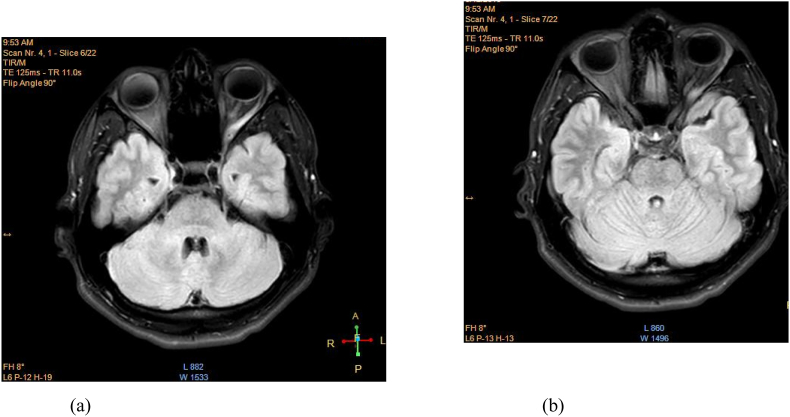
Axial section of MRI brain and orbit showing cystic lesion with myositis of left lateral rectus muscle compressing the optic nerve at the orbital apex.

Laboratory investigations revealed hemoglobin of 14.5 g%, total leucocyte count 7750 cells/mm^3^, neutrophils 65%, lymphocytes 28%, eosinophil 4%, and monocytes 3%, platelets 210000 cells/mm^3^, and random blood sugar 77 gm/dl. Serological test for RA and ANA were non-reactive. Serum Cysticercosis antibody was found to be negative by IgG ELISA. Magnetic resonance imaging (MRI) of the orbit revealed a cystic lesion in the left lateral rectus muscle near to its origin in close proximity compressing the optic nerve at orbital apex (Fig. 2). MRI of brain and paranasal sinus were normal.

Following this the diagnosis of left lateral rectus cysticercosis with compressive optic neuropathy was made. He was given prednisolone (60 mg PO OD) for 1 month in tapering dose along with pantoprazole (40 mg OD), and albendazole (400 mg PO BD for 4 weeks).

After treatment patient showed alleviation of symptoms, the vision in LE was restored to 6/6 with disappearance of RAPD by 1 week. However, diplopia was still present with restricted left abduction movement. On follow-up after one month, there was improvement in left eye esotropia (10 PD on prism bar cover test) but binocular horizontal diplopia was persistent with only limited improvement in the LE abduction deficit. Visual acuity (OU) was 6/6 with normal pupils and no RAPD, color vision and contrast sensitivity were normal in both eyes.

## 3 Discussion

Cysticercosis is well-known zoonotic disease caused by the parasite of *Taenia* species. Orbital and adnexal cysticercosis can present with various clinical presentation [4]. Extraocular muscles are one of the most common lodgment sites of encysted larvae. Taenia pierce into the human tissues, including the extraocular muscle, forming larval cysts (cysticercus) with scolex, which degenerate over time and may release cytotoxins (classical parenchymal type) [5]. Regarding the commonest extraocular muscle site for the lodgment of cyst variable findings are noted and the cyst may lodge in any of the extraocular muscles [1]. In our case cyst was lodged in the left lateral rectus muscle.

Orbital myocysticercosis often mimics various eye pathologies like as isolated nerve palsy, orbital pseudotumor, orbital cellulitis, superior orbital fissure syndrome etc [2,6]. The clinical manifestations can often lead to a misdiagnosis of orbital inflammatory disease especially when the cyst is missed on imaging. Likewise cases of left lateral rectus myocysticercosis was found to masquerade as exotropic duane's retraction syndrome (DRS) (type IIb) with normal visual acuity in affected eye [5,7]. MRI image in our case was also having left lateral rectus myocysticercosis but wasn't mimicking DRS rather showed some unique features mimicking orbital apex syndrome having features like optic neuropathy along with 6th cranial nerve involvement.

Orbital apex syndrome may present with multiple cranial nerve involvement pupillary involvement, optic neuropathy even with decreased corneal sensations and periorbital sensations [8]. The differential diagnosis of orbital apex syndrome include tolosa hunt syndrome, thyroid orbitopathy, mucormycosis, herpes zoster, carotico-cavernous fistula, cavernous sinus thrombosis [8]. In our case, MRI orbit revealed cysticercosis of lateral rectus at orbital apex. Cystic lesion on lateral rectus was located near to the orbital apex close to the muscle origin where it compressed the optic nerve resulting compressive optic neuropathy. Similar to our case, a rare presentation of extraocular muscle cysticercosis near the optic foramen causing optic foramen syndrome resulting into optic nerve compression and vision loss which was managed by emergency optic nerve decompression and cyst excision [2].

Acute vision loss in a case of orbital cysticerosis is mainly due to compressive optic neuropathy due to cystic lesion of extra ocular muscle or by direct invasion of the optic nerve or as a result of compression from the adjacent cyst in intra conal region [2]. But direct optic nerve invasion of cysticercosis is a very rare entity (10)*.* Orbital apex syndrome secondary to myocysticercosis was a diagnostic challenge in the present situation due to its rarity. To the best of our knowledge there is only one other case of orbital apex syndrome due to myocysticersosis till date [8].

The serological tests used for specific diagnosis of cysticercosis are indirect hemagglutination, indirect immunofluorescence, and immune electrophoresis such as ELISA serology is the most sensitive. FLAIR images in MRI scan have maximal rates of scolex detection hence should better to considered as diagnostic investigation over ultrasonography (USG) and computed tomography (CT) in orbital cysticercosis and neurocysticercosis [6]. Positive test results from a serum enzyme-linked immunosorbent assay (ELISA) for anticysticercal antibodies help confirm the diagnosis; however, negative test results do not exclude cysticercosis [9]. In our case cysticercosis IgG ELISA was negative whereas MRI revealed myocysticercosis of lateral rectus.

Albendazole along with steroid is used as the first-line treatment because of its higher cure rates and easy availability. Surgical intervention can be performed in patients that are non-responsive to treatment, in those where the cyst is at surgically accessible location (superior extraconal space involving SR-LPS complex, subconjunctival and eyelid cysticercosis) [[1], [2]]. Surgical decompression is also a method of choice in case of compressive optic neuropathy with acute vision loss [2].

Some residual deficits are noted even after resolution of the condition which can manifest as diplopia (13.1%), strabismus (8.19%),and ocular movement restriction (18.03%) [10]. In our case, residual diplopia and residual abduction and mild adduction deficit were present on 4 week post medical treatment [10]. Prism glasses can be employed for correction of residual strabismus and for reliving the diplopia.

## 4 Conclusion

Orbital apex syndrome secondary to myocysticercosis is rare but can result in visual compromise if not managed timely. MRI can be the best imaging tool for prompt diagnosis and to rule out other differentials. Cysticerci can lodge themselves in any part of the ocular and extra ocular tissue. Medical management in the form of oral Albendazole combined with oral steroids is the treatment of choice followed by surgical treatment (cyst excision or orbital decompression) to avoid permanent vision loss.

## Provenance and peer review

Not commissioned, externally peer-reviewed.

## Funding

No funding was received for the study.

## Ethical approval

None.

## Consent

Written informed consent was obtained from the patient.

## Registration of research studies

Name of the registry: None
Unique Identifying number or registration ID: None
Hyperlink to your specific registration (must be publicly accessible and will be checked):

## Data availability statement

All the required information is in manuscript itself.

## Authors’ contribution

BK and SS wrote the original manuscript, reviewed, and edited the manuscript. SJS reviewed and edited the original manuscript. SS, BK, and GBS reviewed and were in charge of the case.

## Guarantor

Bipin Koirala.

## Declaration of competing interest

Authors have no conflicts of interest to declare.
